# Exploring factors affecting nurses’ job satisfaction at a regional hospital in Namibia

**DOI:** 10.4102/curationis.v49i1.2830

**Published:** 2026-04-29

**Authors:** Matias M. Kalomo, Daniel O. Ashipala, Alice Lifalaza, Epafras Anyolo, Julia Amadhila

**Affiliations:** 1 Department of General Nursing Sciences, Faculty of Health Sciences and Veterinary Medicine, University of Namibia, Windhoek, Namibia; 2 Department of General Nursing Science, Faculty of Health Sciences and Veterinary Medicine, University of Namibia, Rundu, Namibia; 3 Department of General Nursing Science, Faculty of Health Sciences and Veterinary Medicine, University of Namibia, Oshakati, Namibia

**Keywords:** human, hospitals, health care sector, job satisfaction, motivation, Namibia

## Abstract

**Background:**

Job satisfaction encapsulates the degree to which employees perceive fulfilment in various facets of their work, including the stability of their position, opportunities for professional development and confidence in their capability to execute tasks successfully. In Namibia, a decline in job satisfaction has been observed with nurses leaving hospitals at alarming rates, yet limited research exists on the factors influencing their job satisfaction. This study was conducted at Rundu Intermediate Hospital between June 2024 and September 2024.

**Objectives:**

The study explored factors affecting job satisfaction among nurses at Rundu Intermediate Hospital in the Kavango East Region, Namibia.

**Method:**

A qualitative, exploratory, descriptive and contextual design was used. Fifteen nurses from various departments were selected through non-probability convenience sampling. Data were collected via semi-structured interviews, audio-recorded, transcribed verbatim and analysed thematically.

**Results:**

Nurses’ job satisfaction is closely linked to personal fulfilment, professional growth and contributions to patient care. Objective factors such as promotion, pay and recognition further shape satisfaction levels. Major challenges include staff shortages, resource constraints and lack of support.

**Conclusion:**

Hospital management should implement strategies that reduce workload, enhance recognition and create opportunities for professional development to improve job satisfaction and retention.

**Contribution:**

The study provides context-specific insights into factors influencing nurses’ job satisfaction in Namibia and proposes management interventions to strengthen morale, support professional growth and improve the quality-of-patient care.

## Introduction

Globally, job satisfaction has been a critical issue for healthcare organisations in recent years, particularly in nursing (Lucas et al. 2025). Yunike et al. (2023) indicated that in order to enhance nurses’ job satisfaction and ultimately improve the quality of patient care, it is crucial to examine the factors that impact their overall work experience (Ayalew & Workineh 2019). In a constantly challenging environment, healthcare systems are expected to achieve the often competing aims of improving public health (Frisicale et al. 2019). Nurses have an important key role to play in the achievement of Sustainable Development Goal (SDG) three, which includes health promotion, chronic disease management of aging population by addressing both their physical and psychosocial dimensions of ageing as well teaching the patients how to prevent illness (Das et al. 2021). They are also expected to deliver empathetic patient care while facing a highly stressful environment characterised by limited resources and heavy workloads (De Oliveira et al. 2019). Job satisfaction of nurses is extremely important due to their professional tasks being related to the care of patients (Dziedzic et al. 2023). Hospitals with satisfied nurses are also more likely to good patient experience, staff and partnerships, which helps them succeed and grow.

Poor job satisfaction among nurses is not a new phenomenon (Mere et al. 2023); research shows that just 23% of Israeli nurses are happy, followed by 58% in Nepal, 61% in Indonesia and 63% in Iran (Javanmardnejad et al. 2021). In North West Ethiopia, nurses’ job satisfaction is just 44% (Ayalew & Workineh 2019), while 72% of nurses in Kwale and Kilifi counties in Kenya noted low levels of job satisfaction (Tengah & Otieno 2019). A study conducted by Kassa and Bedada (2021) in Botswana showed that the overall job satisfaction of nurse anaesthetists was only 36%. In the Namibian context, Shalonda (2019) revealed that most nurses were dissatisfied with their salaries (60%), working conditions (53%) and the chance for promotion and advancement opportunities (66%). Investigating the intricate dynamics of nurses’ job satisfaction is thus not only vital for their professional well-being but also holds the key to ensuring the provision of optimal patient care within healthcare systems (Flaubert et al. 2021).

This study investigated the factors influencing job satisfaction at Rundu Intermediate Hospital in the Kavango East Region of Namibia, as there has been limited research in the Namibian context. The 2013 Presidential Commission of Enquiry by the Ministry of Health and Social Services (MoHSS) highlighted growing concerns about the high rate of nurse attrition in public hospitals, which threatens healthcare delivery (MoHSS 2013). A recent study conducted at a district hospital in Namibia found that heavy workloads, inadequate support and poor working conditions significantly undermine nurse satisfaction and contribute to turnover (Ashipala, Albanus & Nghole 2023). Broader occupational stress research confirms these findings, underscoring the need to address burnout to retain professionals in demanding healthcare environments. Literature documents four factors impacting on job satisfaction of employees, which includes work environment, salary and benefits, career development and leadership style (Wang 2024). Additionally, job satisfaction is also related to many other different factors such as job characteristics that are consistent with expectations, job advancement opportunities, compensation that is appropriate to the job, good relationships with coworkers and supervisors just among others (Sinpattanaphong 2017).

### Research purpose

The purpose of this study was to explore and describe the factors affecting nurses’ job satisfaction at Rundu Intermediate Hospital in the Kavango East Region of Namibia. The findings are expected to guide the MoHSS in developing evidence-based strategies to enhance job satisfaction, strengthen retention and promote a healthier work environment for nurses. In addition, the results will assist healthcare human resource managers (HRMs) across Namibia to design and implement practical strategies to better support nurses, improve morale and reduce turnover.

## Research methods and design

### Design

A qualitative study with an exploratory, descriptive and contextual design was employed to answer the research questions. The exploratory approach enabled a deep understanding of nurses’ experiences regarding job satisfaction; the descriptive design facilitated the clear presentation of findings through coding and theming of the participants’ perspectives (Rutberg & Bouikidis 2018), and the contextual design allowed data to be collected within the participants’ work environment, ensuring relevant and reliable insights.

### Setting

The study was conducted at Rundu Intermediate Hospital in Rundu town, Kavango East Region, Namibia. The hospital has 167 registered nurses and 157 enrolled nurses, who work 12-h shifts on a rotating schedule. With a total bed capacity of 519, the hospital includes units such as casualty, high care, paediatrics, maternity, surgical, prem, tuberculosis and male and female wards. The hospital serves patients from the Zambezi, Kavango East and Kavango West regions. Fifteen nurses who met the inclusion criteria from various units participated in the study.

#### Population and sampling strategy

The population of this study consisted of all nurses employed at Rundu Intermediate Hospital in the Kavango East Region, Namibia. At the time of the study, 324 nurses registered and enrolled nurses were responsible for providing direct patient care. The target population included nurses who were available and willing to participate in the study. A non-probability, snowball recruitment strategy was employed to select 15 nurses for the interviews. Access to participants was facilitated through the Nursing Department supervisors, who informed eligible nurses about the study and provided their contact details. Appointments were then scheduled with each participant.

### Data collection methods

Data were collected through semi-structured interviews between June 2024 and September 2024. An interview guide was developed based on the study objectives and existing literature, which was used to explore factors affecting nurses’ job satisfaction among nurses at Rundu Intermediate Hospital, Kavango East, Namibia. English was used as all participants were proficient due to their academic and professional backgrounds, ensuring clarity and authenticity of the data collected. The researcher obtained permission to conduct the study from the HRM of Rundu Intermediate Hospital, who also granted access to the hospital and assisted in facilitating contact with participants. The researcher had no prior personal relationships with the participants that could influence their responses. To prevent coercion, participants were fully informed about the purpose of the study, the voluntary nature of participation and their right to withdraw or decline to answer any questions at any time. Written informed consent was obtained from all participants prior to the interviews, and confidentiality and anonymity were emphasised, with assurances that all information would be securely stored and used solely for research purposes.

Interviews were conducted at times and locations selected by the participants, typically within hospital offices or quiet areas near their departments, providing a conducive environment for open discussion without interruptions. Field notes were taken to capture non-verbal cues and relevant environmental observations, and the interviews were audio-recorded with the participants’ permission. Each interview lasted approximately 30 min – 45 min.

The researcher posed the following core open-ended questions, with follow-up probes based on the participants’ responses: (1) *How would you describe your level of job satisfaction in your current role?;* (2) *What factors in your workplace contribute to or hinder your job satisfaction?;* (3) *How do recognition, workload, remuneration and professional development opportunities influence your motivation and engagement?* and (4) *What strategies or interventions do you think hospital management could implement to improve nurses’ job satisfaction and retention?*

### Data analysis

Data saturation was reached by the 15th participant, indicating that no new information would emerge from additional interviews. Raw data from the audio-recordings were transcribed verbatim before they were analysed using Braun and Clarke (2019) six steps of data analysis: (1) familiarisation with the data by rereading interview transcripts along with field notes and listening to the recordings multiple times to immerse oneself in the data; (2) generating initial codes in order to describe the content and identify and provide a label for features of the data that were relevant to the research question; (3) searching for themes in the data of all the interview transcripts by reviewing the coded data to identify areas of similarities and overlap; (4) reviewing potential themes by checking them against the organised extracts of the data and if the themes work in relation to the data; (5) defining and naming themes by summarising each theme in a few sentences to produce a coherent and overall account of the data and (6) producing a report by writing a clear, convincing and complex description of the data based on the data analysis. The data were independently analysed by two analysts – an independent coder and the researcher. Each analyst conducted their own thematic analysis, and any discrepancies or potential biases were discussed in detail. Decisions were reached through consensus to ensure a thorough and unbiased interpretation of the data. This multilayered approach helped to enhance the validity and credibility of the data analysis process. After initial coding, the researcher met with the co-coder (the data analyst) to discuss the findings from both of their perspectives. There was general agreement on key issues surrounding the codes, subthemes and themes. The final wording of the findings did not exactly match the initial findings or the findings from the co-coder. However, the key aspects were retained, and these findings matched most of the subthemes from the co-coder. Any differences can be attributed to the researcher’s condensing of the themes and further aligning them to the data. The independent coder was selected because of a Doctorate in Nursing education, where a qualitative interview method was utilised.

### Measures to ensure trustworthiness

The four principles framework of Lincoln and Guba (1985) was used to ensure the trustworthiness of this study. The researcher aimed to guarantee the trustworthiness of the study through confirmability, credibility, transferability and dependability. Confirmability was supported through the use of multiple data collection methods, such as audio recordings and detailed field notes. This triangulation of data sources ensured that the findings were firmly grounded in participants’ experiences. In addition, the researcher kept an audit trail documenting decisions and procedures throughout the study, enabling verification of the findings. Credibility was established by maintaining prolonged engagement with participants, spending extended time interacting with them in their workplace. After the initial data collection and analysis, the researcher returned to the participants to present the findings derived from their data. Participants were asked to review these findings and provide feedback on whether they accurately represented their experiences or perceptions. The researcher sought clarification, additional insights or corrections from the participants based on their feedback, thereby enhancing the trustworthiness of the interpretation. Transferability was strengthened by providing thick descriptions of the research context, participants and methodology, enabling other researchers to assess the relevance of the findings to different settings. Through detailed accounts of participants’ experiences, the study offers insights into how the findings may be applied to other contexts. Dependability was ensured through the involvement of an independent co-coder who reviewed the data and findings, offering an external perspective on the analysis. A detailed account of the study design and methods was also provided, promoting transparency in the research process.

### Ethical considerations

Prior to data collection, ethical approval for the study was obtained from the School of Nursing Research and Ethics Committee (SoNREC) at the University of Namibia (SoNREC 99/2023). Permission to conduct the study was also granted by the Ministry of Health and Social Services Institutional Research Review Board (Reference number: 22/7/7/2). Confidentiality and anonymity were strictly maintained throughout the study. Participants’ names were replaced with codes, and all information was securely stored on a password-protected computer accessible only to the researcher and study supervisors. Participants were informed that they would not receive direct personal benefits, but that the findings of the study were intended to inform strategies to enhance nurses’ job satisfaction, which could positively impact the work environment at Rundu Intermediate Hospital. The study adhered to the principle of non-maleficence, as no interventions were conducted that could potentially harm participants.

## Results

### Demographic profile of participants

The study included 15 nurses (*n* = 15) aged between 25 years and 52 years, with the majority falling within their late twenties to mid-thirties. The participants comprised nine females and six males, reflecting a slightly higher representation of females. In terms of qualifications, most were registered nurses (*n* = 11), while a smaller group were enrolled nurses (*n* = 4). Their years of working experience ranged from 2 years to 25 years, indicating a mix of junior, mid-level and highly experienced nurses. This diversity in age, gender, qualification and professional experience provided a broad perspective on the study topic. Table 1 shows the participants’ demographic data.

**TABLE 1:** Demographic characteristics of participants.

Participant codes	Age (years)	Gender	Nursing qualification	Working experience (years)
P1	36	Female	Registered	8
P2	32	Female	Registered	5
P3	32	Female	Enrolled	6
P4	33	Male	Enrolled	11
P5	30	Male	Enrolled	7
P6	30	Male	Registered	3
P7	26	Male	Registered	3
P8	52	Female	Registered	25
P9	26	Male	Registered	2
P10	26	Female	Registered	3
P11	25	Female	Enrolled	3
P12	37	Female	Registered	10
P13	39	Female	Registered	14
P14	32	Female	Registered	3
P15	36	Male	Registered	5

P, participant.

Table 2 lists four themes and 14 subthemes based on the nurses’ rich insights regarding factors that contribute to job satisfaction or dissatisfaction.

**TABLE 2:** Themes and subthemes that emerged from analysis.

Themes	Subthemes
1. Understanding of job satisfaction	1.1. Personal fulfilment
2. Factors affecting job satisfaction	2.1. Opportunities for career advancement 2.2. Team work 2.3. Better wages 2.4. Coworker relationships
3. Factors contributing to job dissatisfaction	3.1. Heavy workload 3.2. Shortage of resources 3.3. Lack of recognition 3.4. Lack of opportunities for continuous professional development
4. Recommendations to improve job satisfaction among nurses	4.1. Review nurses’ salary grading 4.2. Training and development opportunities 4.3. Ensure availability of equipment and supplies 4.4. Management support

### Theme 1: Understanding of job satisfaction

This theme captures the participants’ perceptions regarding job satisfaction; they described it as a sense of personal fulfilment, emotional well-being and accomplishment in their work. They emphasised that job satisfaction goes beyond salary or career advancement to include feeling valued, supported and able to perform their duties effectively.

#### Subtheme 1.1: Personal fulfilment

This subtheme comprises participants understanding concept definition of job satisfaction. Numerous participants understood the term ‘job satisfaction’ as meaning an individual’s positive or negative emotional response towards their job role and level of happiness derived from the job:

‘When an employee feels good or stable about their job and they also feel they can also grow in their job or career; also, this relates to personal life.’ (P4, 33 years, male)

### Themes 2: Factors affecting job satisfaction

This theme relates to which factors contribute to participants’ job satisfaction and their effect on patient care, with the following subthemes: Opportunities for career advancement, team work, better wages and coworker relationships.

#### Subtheme 2.1: Opportunities for career advancement

Participants highlighted that opportunities for career advancement are a contributing factor to nurses’ job satisfaction. Participants also stated that they are able to sharpen their skills and knowledge in nursing at their jobs as the hospital is a training hospital.

#### Subtheme 2.2: Team work

Participants emphasised that collaboration with other health workers promotes patient-centred care and reduces stress among nurses, leading to increased job satisfaction. They highlighted that effective teamwork fosters mutual support, improves efficiency and creates a positive work environment:

‘We share the information on how to treat the patient and how to maintain the well-being of the patient as a teamwork.’ (P8, 52 years, female)

#### Subtheme 2.3: Better wages

Participants highlighted that fair salaries enable them to support their families, reduce financial stress and remain motivated to provide quality patient care. They emphasised that adequate pay not only meets their basic needs but also serves as recognition of their hard work, which fosters loyalty, commitment and overall job satisfaction:

‘A good salary makes me satisfied as a nurse as it boosts my work morale and life, because if I am paid a salary that can cater for my basic needs it boosts my energy and confidence and at the end the day, I will even give proper nursing care since I do not have worry when it comes to finances.’ (P7, 30 years, male)

#### Subtheme 2.4: Coworker relationships

Participants highlighted that inter-professional respect improves job satisfaction and well-being. Positive relationships and teamwork foster trust, reduce stress and enhance patient care. Participants added that mutual support among colleagues creates a healthy work environment, while poor relationships are demotivating and affect overall performance:

‘The staff relationships whereby there is mutual respect and dignity and as well me providing nursing care and my patients gets well afterwards it really makes me feel satisfied.’ (P7, 26 years, male)

### Theme 3: Factors contributing to job dissatisfaction

This theme explores the various factors participants have experienced that limit their job satisfaction. The key aspects discussed under this theme include heavy workload, shortage of equipment, lack of recognition and limited opportunities for continuous professional development.

#### Subtheme 3.1: Heavy workload

Participants mentioned that despite satisfying aspects of their workplace, an imbalanced nurse-to-patient ratio significantly increases their work burden. This heavy workload leads to fatigue, stress and burnout, making the profession less appealing and reducing interest in pursuing a long-term nursing career. Nurses reported that excessive responsibilities often limit their ability to provide quality patient care, which further impacts their job satisfaction and overall well-being:

‘I want to render that proper care but then I cannot give to all of them because there are a lot of patients, so I have to do here and there so thus I am not satisfied.’ (P10, 26 years, female)

#### Subtheme 3.2: Shortage of equipment

Nurses reported that a shortage of medical equipment frustrates them by limiting their ability to provide quality care. This shortage complicates achieving work goals and negatively impacts job satisfaction due to compromised service delivery. Participants further noted that a lack of essential tools not only affects their efficiency but can also delay treatment, increase patient risks and create additional stress in the workplace:

‘If we do not have the necessary resources or for us to progress to the next level it makes me feel unsatisfied because one would not achieve their goals and the work becomes complicated.’ (P4, 33 years, female)

#### Subtheme 3.3: Lack of recognition

The nurses commented that they are the beating heart of medical teams, delivering care on regular basis, making judgements and providing comfort during times of crisis. However, many times their efforts are ignored, and achievements may go unnoticed or unacknowledged by supervisors, and the nurses are not aware of the method that was utilised to promote those that are recognised:

‘We are not appraised in areas that we did well, and those that are recognised we do not know how they were selected by the management that they are hard workers, for him or her to deserve to be recognised.’ (P14, 32 years, female)

#### Subtheme 3.4: Lack of opportunities for continuous professional development

Nurses stated that in-service training is a major concern in the hospital, as nurses are not frequently given opportunities for further training and not everyone has an equal chance to attend. They emphasised that limited access to continuous professional development hinders skill improvement, reduces confidence and affects the quality of care delivered. Some participants expressed frustration, noting that unequal opportunities create feelings of neglect and demotivation among staff:

‘At the clinic I was working I got exposed to a lot of programmes whereby here in the hospital it is limited and not everybody has access to such programmes; so to say programmes for training for TB, IMCI [*Integrated Management of Childhood Illness]* and HIV, so here in the hospital not every nurse has a chance to be sent for training.’ (P14, 32 years, female)

### Theme 4: Recommendations to improve job satisfaction among nurses

This theme provides suggestions for promoting job satisfaction among nurses at Rundu Intermediate Hospital. The subthemes include the need to recruit more nurses to reduce workload, to review and improve nurses’ salaries to enhance motivation and to increase opportunities for in-service training in various aspects of nursing care to strengthen skills and confidence.

#### Subtheme 4.1: Review nurses’ salary grading

Participants stated that although they are being paid, there is a need to review and increase their salaries to boost morale and motivation in the workplace. Participants emphasised that better compensation would help retain staff, reduce financial stress and enhance commitment to providing quality patient care:

‘Government should review the salary grading of enrolled nurses at least to push it to grade 9 not grade 10; like that is too little for us.’ (P5, 30 years, male)

#### Subtheme 4.2: Training and development opportunities

Participants suggested further training on patient care and current nursing guidelines through in-service programmes to enhance their skills and knowledge. They also emphasised that regular training would improve confidence, ensure up-to-date practices and lead to better quality care for patients:

‘We need proper and fresh in-service training on the latest update information about how to take care of the patient so that we can provide proper nursing care based on the guidelines and policies.’ (P8, 52 years, female)

#### Subtheme 4.3: Ensure availability of equipment and supplies

Participants proposed that hospital management should procure quality equipment and necessary supplies to support patient care and ensure accurate results. In addition, they emphasised that having the right tools reduces frustration, improves efficiency and enhances their ability to provide safe and effective care, which in turn promotes morale and overall job satisfaction:

‘We need equipment which we can use to monitor patients for the services to improve and nurses will even be happy because they are not going to struggle.’ (P1, 36 years, female)

‘We need good quality instruments that can give proper results so that we do not end up losing our patients as result of equipment.’ (P8, 52 years, female)

#### Subtheme 4.4: Management support

Participants suggested that hospital management should regularly visit units to identify issues affecting nurses and address them promptly. They emphasised that active management support would improve nurses’ job satisfaction, foster a supportive work environment and ensure the delivery of high-quality patient care:

‘The management should come on the ground to see how we are working, ask challenges we are facing and familiarise themselves with daily operations.’ (P6, 30 years, male)

‘I think the management should not only be in the offices; they should come and do daily inspections because sometimes even if we raise a complaint that there are too few staff in the unit, they just say a shortage of nurses is everywhere.’ (P2, 32 years, female)

## Discussion

The participants comprised nine females and six males, reflecting a slightly higher representation of females. This gender disparity between males and females can be attributes to the fact that the nursing profession is viewed as a female profession. In terms of qualifications, most were registered nurses, while a smaller group were enrolled nurses. Their years of working experience ranged from 2 years to 25 years, indicating a mix of junior, mid-level and highly experienced nurses. This diversity in age, gender, qualification and professional experience provided a broad perspective on the study topic.

This study explored and described the factors that affect job satisfaction among nurses at Rundu Intermediate Hospital in Kavango East Region, Namibia and provided recommendations to improve satisfaction, promote nurse retention and enhance the quality of nursing care. The participants demonstrated an acceptable understanding of the concept of job satisfaction, defining it as feelings of stability in one’s role and the ability of the job to fulfil personal needs. This aligns with Tomaszewska et al. (2024), who described job satisfaction as a positive attitude of employees towards their duties, work environment and colleagues.

Several factors were described as contributing to nurses’ satisfaction, including opportunities for career advancement, adequate remuneration, teamwork and positive relationships with coworkers. These findings are consistent with Bragadóttir et al. (2023), who found a positive relationship between nursing teamwork and job satisfaction, highlighting teamwork as essential for patient safety and quality care. Similarly, Fox, McAllum and Ginoux (2023) emphasised that team care involving sharing, support and compassionate leadership can alleviate healthcare workers’ stress and promote well-being. Participants also highlighted the importance of a supportive work environment, including assistance from coworkers and management, which boosts morale. This is supported by O’Hara et al. (2019), who found that supportive leadership is a primary contributor to work satisfaction among millennial nurses. Participants further noted that job satisfaction is enhanced when nurses collaborate, share ideas for patient care and receive timely salaries. These findings echo those of Demeubaeva et al. (2024), who reported that team coherence, trust and a positive environment influence nurses’ satisfaction, while Akinwale and George (2020) identified salary and career advancement as top priorities.

Moreover, participants reported that excessive workloads, often due to a high nurse-to-patient ratio, negatively affects their job satisfaction and interest in the profession. These findings are in line with those of Garcia (2023), while Ashipala and Nghole (2022) reported that patient overcrowding in Namibia leads to emotional exhaustion, compromising nurses’ ability to deliver quality care. Lack of recognition was another significant factor affecting satisfaction, with nurses expressing that supervisors and management rarely acknowledge their efforts, making them feel undervalued. These findings support those of Garcia (2023), who highlighted that insufficient recognition leads to burnout, weakens professional identity and diminishes the perceived value of care. Al Ahmari et al. (2023) also noted that recognition and empowerment are essential for retaining nurses and increasing job satisfaction.

Participants further indicated that inadequate medical equipment, such as blood pressure machines and thermometers, hinders the provision of quality care. This correlates with a study by Moyimane, Matlala and Kekana (2017), who reported that equipment shortages negatively affect patient care, hospitals and the nursing profession. Critical shortages were attributed by the nurses to malfunctioning equipment, poor maintenance and budgetary constraints. They also highlighted that opportunities for further education and training would enhance their competence and job satisfaction. This is as per Suprapto, Mulat and Lalla (2021), who emphasised that professional development improves nurses’ dedication and commitment, while Holmberg, Caro and Sobis (2018) suggested that ward managers should establish clinical ladder programmes to identify and support nurses’ continuous professional development.

The participants strongly recommended several measures to enhance job satisfaction and the work environment, including hiring additional staff to reduce workload, improving patient care and ensuring adequate staffing to meet unit goals. These results corroborate with a study conducted by Albashayreh et al. (2019), who emphasised that healthcare leaders are encouraged to develop policies promoting staffing, resources, wages, benefits, career advancement and nurse participation in hospital affairs. Participants also stressed the need for management support to identify and address nurses’ challenges. Liu et al. (2023) similarly recommended that nursing managers implement reasonable incentive systems and provide organisational support to reduce turnover intentions. Lastly, the findings indicate that nurses’ job satisfaction is influenced by a combination of personal, professional and organisational factors, and addressing these factors holistically is essential for improving retention, morale and the quality of patient care.

### Practical implications

The findings of this study provide valuable insights for hospital management and HRMs at Rundu Intermediate Hospital and other hospitals in Namibia. To improve nurses’ job satisfaction, hospital management should enhance recognition and professional development by providing opportunities for promotion, career growth and continuous training. Moreover, remuneration and benefits should be reviewed to ensure they are fair and comparable to similar positions in other healthcare settings. Reducing nurses’ workloads through adequate staffing and optimised shift schedules can help prevent burnout and improve their work–life balance. In addition, strengthening leadership and management support through training in transformational and supportive leadership can foster positive communication, trust and morale among staff. Promoting teamwork and a positive organisational culture through team-building initiatives and peer support programmes can further enhance job satisfaction. Additionally, implementing employee well-being programmes to address stress, burnout and occupational health risks will support nurses’ mental health and overall well-being. Collectively, these interventions could increase staff retention, boost motivation and improve the quality of patient care while creating a more supportive and positive work environment for nurses.

### Limitations and areas for further research

The findings are limited to nurses working at the Rundu Intermediate Hospital, however, and may not reflect experiences in other healthcare settings with different contexts. The use of convenience sampling may also have influenced the range of perspectives captured, as not all nurses may have been represented. Nevertheless, the study revealed important areas for further exploration, including how nurse-to-patient ratios affect job satisfaction and how experiences of job satisfaction may differ across nursing departments.

### Recommendations

Based on the findings of this study, the following recommendations are made:

#### Creating a conducive working environment

Management should prioritise creating a supportive and conducive working environment to ensure that nurses feel happy, fulfilled and engaged in their roles. A positive work environment reduces stress, enhances motivation and promotes overall job satisfaction. This can be achieved by promoting mutual respect, open communication and collaborative teamwork, which will foster a sense of belonging and professional pride among nurses.

#### Adequate staffing and workload management

Ensuring adequate nurse-to-patient ratios in all units and on each shift is essential to prevent excessive workload and burnout. Overburdened nurses may experience fatigue, reduced performance and decreased satisfaction. Proper staffing allows nurses to provide high-quality care, meet patient needs efficiently and maintain a balanced work–life schedule, which directly influences retention and satisfaction.

#### Provision of high-quality equipment and supplies

Maintaining sufficient medical equipment and supplies, such as blood pressure machines, thermometers and other essential tools, will support nurses in performing their duties effectively. Shortages of equipment increase stress, compromise patient care and diminish job satisfaction. Ensuring that a hospital is consistently stocked with functional and high-quality resources will enable nurses to work efficiently and safely.

#### Recognition and reward systems

A clear system of recognising high-performing nurses should be established through awards, salary increases and promotional opportunities. Recognition fosters a sense of value, motivates staff and encourages professional growth. Reward systems not only increase individual satisfaction but also promote a healthy competitive spirit within nursing units, enhancing overall morale and engagement.

#### Professional development and career advancement

Supporting nurses’ professional growth through opportunities for further studies, in-service training, workshops and clinical skill development is vital. Continuous learning improves competence, confidence and job satisfaction. Additionally, structured pathways for career advancement, promotions and leadership development empower nurses to achieve personal and professional goals.

#### Empowering nurses through participation in policy development

Allowing nurses to contribute ideas to hospital unit policies and protocols will empower them and foster a sense of ownership and appreciation. Participation in decision-making strengthens engagement, ensures that policies are practical and relevant and enhances job satisfaction by making nurses feel heard and valued within the organisation.

#### Fostering teamwork and mentorship

Encouraging strong teamwork, collaboration and peer support within nursing units will enhance trust, communication and cooperation. Mentorship programmes for junior or less experienced nurses can provide guidance, reduce stress and improve confidence. Positive interactions among colleagues are closely linked to higher job satisfaction and improved patient care outcomes.

#### Fair compensation and incentives

Timely payment of salaries, competitive wages and additional incentives are crucial for nurse motivation and job satisfaction. Financial recognition reinforces the value of nurses’ work and encourages commitment to the organisation. Policies addressing fair remuneration contribute to retention and reduce turnover intention.

#### Monitoring and addressing workplace challenges

Hospital management should establish systems to identify workplace challenges, gather feedback and implement interventions to address occupational stress, professional recognition, workload issues and opportunities for career growth. Active management of these factors will ensure that nurses remain satisfied, motivated and capable of delivering high-quality care to patients.

## Conclusion

The study revealed multiple factors affecting job satisfaction among nurses at Rundu Intermediate Hospital, including workload, lack of recognition, insufficient resources, limited career advancement opportunities, inadequate professional development and the need for supportive management and teamwork. Nurses reported that excessive patient loads, shortages of essential medical equipment and minimal acknowledgement of their efforts hinder their motivation and satisfaction, while positive relationships with colleagues, opportunities for skill development and supportive leadership enhance their engagement. It is imperative that hospital management and relevant stakeholders address these factors to improve nurses’ job satisfaction, promote staff retention and enhance the quality of patient care. The findings of this study may contribute to the development of policies and interventions that foster a supportive work environment, provide adequate resources and encourage professional growth, ultimately strengthening nursing practice and improving health outcomes in the region. By addressing these factors, healthcare leaders can ensure a motivated, competent and satisfied nursing workforce capable of delivering high-quality and safe patient care.
